# Efficacy of repetitive transcranial magnetic stimulation in the prevention of relapse of depression: study protocol for a randomized controlled trial

**DOI:** 10.1186/1745-6215-14-338

**Published:** 2013-10-17

**Authors:** Huaning Wang, Yunyun Xue, Yunchun Chen, Ruiguo Zhang, Huaihai Wang, Yahong Zhang, Jingli Gan, Liyi Zhang, Qingrong Tan

**Affiliations:** 1 Department of Psychiatry, Xijing Hospital, The Fourth Military Medical University, 15 Changle Road, 710032, Xi’an, China; 2 Department of Psychiatry, 91 Hospital of PLA, 239 Gongye Road, 454003, Jiao’zuo, China; 3 Department of Psychiatry, 102 Hospital of PLA, 55 Heping Road, Chang’zhou 213003, China

**Keywords:** Repetitive transcranial magnetic stimulation (rTMS), Depression, Relapse

## Abstract

**Background:**

Depression is a chronic illness that generally requires lifelong therapy. Repetitive transcranial magnetic stimulation (rTMS) is a noninvasive technique with few side effects that has been reported to be useful in the treatment of depression. However, no studies to date have evaluated in a randomized controlled trial (RCT) the efficacy of rTMS for maintenance treatment of depression.

**Methods/design:**

In this article, we report the design and protocol of a randomized, single-blind, placebo-controlled, parallel-group, multicenter study in China to evaluate the efficacy of rTMS in the prevention of relapse of depressive symptoms. In total, 540 patients, aged 18 to 60 years, diagnosed with depression and experiencing an acute exacerbation of depressive symptoms, will be enrolled. The study will consist of four phases: a screening/tolerability phase of up to 7 days; an open-label, flexible-dose lead-in phase of 8 weeks; an open-label, fixed-dose stabilization phase of 6 weeks; and a single-blind relapse prevention phase of 12 months. During the open-label phase, all patients will be treated with venlafaxine. Remitters with Hamilton Rating Scale for Depression (HAM-D_17_) score ≤7 will be eligible to enter the single-blind phase and will be randomly assigned to one of three groups: group 1 on active rTMS and venlafaxine; group 2 on sham rTMS and venlafaxine; and group 3 on venlafaxine alone. Efficacy will be evaluated during the study using relapse assessment (time between subject randomization to treatment and the first occurrence of relapse). Secondary outcome measures will include: symptom changes, measured by the HAM-D_17_; illness severity changes, measured by the Clinical Global Impression of Severity for Depression (CGI-S-DEP); and changes in subject functioning, assessed with the Personal and Social Performance (PSP)scale. Safety will be assessed throughout the study by monitoring of adverse events, clinical laboratory tests, electrocardiography (ECG), and measurements of vital signs (temperature, pulse, and blood pressure) and weight. Suicidality will be assessed by the Columbia Suicide Severity Rating Scale (C-SSRS).

**Discussion:**

The result of this trial will assess the efficacy of rTMS in the prevention of relapse of symptoms of depression by determining whether rTMS in combination with an antidepressant is more efficacious than the antidepressant alone for maintenance of the clinical response.

**Trial registration:**

ClinicalTrials.gov, NCT01516931

## Background

Major depressive disorder (MDD) is a chronic disorder characterized by recurrent episodes. Although there are an ever-increasing number of antidepressants available to clinicians, most of these do not produce immediate beneficial actions, and adverse effects are common during the early phases of treatment [1]. Furthermore, approximately 30% of patients are treatment-resistant. Therefore, biological treatments, including repetitive transcranial magnetic stimulation (rTMS) and electroconvulsive therapy (ECT), are considered additional options for the management of MDD.

rTMS is a noninvasive technology that uses a pulsed electromagnetic field to modulate neuronal activity in the cortex of the brain [2], and there is growing evidence that it has antidepressant actions. According to most clinical studies and meta-analyses, highfrequency rTMS is more effective than a sham control in the treatment of MDD in the acute stage and during partial remission [3,4]. Some studies have even reported that the efficacy of rTMS and the longitudinal outcomes of rTMS-treated patients are at least as good as those obtained with ECT [5-7].

Due to the recurrent nature of MDD, a crucial part of the treatment and management of depression is the prevention of these recurrences. However, there are only limited data examining the possibility that rTMS can maintain an antidepressant therapeutic effect. Li *et al*. have described seven adults with bipolar depression who responded acutely to rTMS, and were then treated weekly with rTMS for up to 1 year; the results suggested that rTMS could potentially be used as an adjunctive maintenance treatment for at least some patients with bipolar depression [8]. Abraham and O’Brien reported the case of a 45-year-old woman with a longstanding history of treatment-resistant depression, who had responded well to maintenance rTMS given weekly or biweekly for 4 months [9]. O’Reardon *et al*. applied rTMS to ten adults with MDD, with treatment periods ranging from 6 months to 6 years, and session frequencies averaging 1 to 2 per week; this open-label study suggested that maintenance rTMS may be a safe and effective treatment modality for some patients with unipolar depression [10]. However, all of these reports are limited by their open-label nature and small sample size. Clearly, much work is yet to be done in refining the rTMS parameters (intensity, frequency, pattern, and duration) and the optimal strategy for maintenance treatment. Therefore, sham controlled studies are needed to evaluate the effects of maintenance rTMS in the management of depression.

In view of this, we have designed this randomized, controlled, multicenter study to assess the efficacy and safety of rTMS for delaying the time to relapse in Chinese subjects with MDD. We aim to verify whether rTMS can be a clinically important therapy to prevent MDD recurrence and hence improve the quality of life of patients with MDD.

## Methods/design

### Overview

**Figure 1:**
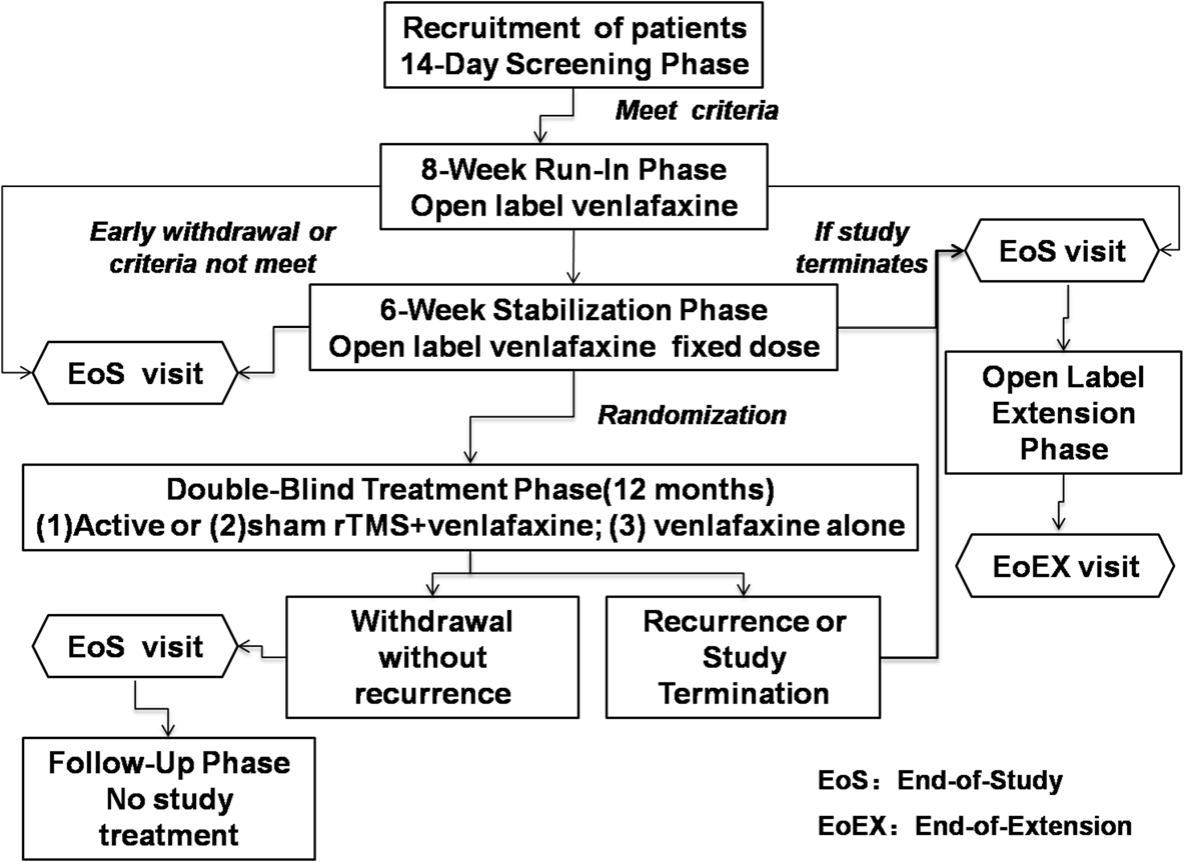
**Flow chart to illustrate the design of the trial.** Enrolment and randomization of the participants, and the follow-up process are shown.

This is a multicenter, randomized, placebo-controlled, single-blind, parallel-group study (Figure 1). This study has been approved by the Ethical Committee of Xijing Hospital, Xian, China, and is registered with the ClinicalTrials.gov database (reference number: NCT01516931). The study will be conducted according to the Declaration of Helsinki, and informed consent will be obtained from each participant.

### Patients and enrolment

#### Inclusion criteria

In total, 540 subjects aged 18 to 60 years, meeting the criteria for depression defined in the Diagnostic and Statistical Manual of Mental Disorders, fourth edition (DSM-IV), and experiencing an acute exacerbation of depression symptoms, having a baseline score of at least 14 points on the 17-item Hamilton Rating Scale for Depression (HAM-D_17_) will be enrolled. Each subject will be asked to spend 8 days as an inpatient at the beginning of the study; willing to and capable of completing the necessary questionnaires; able to take oral medications independently, or have the help and support to do so for the entire length of the study; able to return to the clinical research site for frequent study visits; and considered physically healthy according to screening procedures (including physical examination, electrocardiography (ECG), and clinical laboratory investigations).

#### Exclusion criteria

Patients with any of the following will be excluded: ferromagnetic metallic implants; pacemakers; previous neurosurgery; or a history of seizures, major head trauma, alcoholism, drug addiction, any psychiatric or neurological disorder other than depression and anxiety, psychotic depression, and suicidal propensities.

#### Study process

The main part of the study includes run-in, stabilization, and single-blind phases. The efficacy of rTMS, compared with venlafaxine and placebo, in delaying the time to relapse of symptoms of depression will be evaluated in the single-blind phase, after initial symptom stabilization in the run-in/stabilization phases (Figure 1).

Subjects (inpatients or outpatients) will be evaluated for study eligibility based on prospectively defined inclusion and exclusion criteria during a screening phase of up to 14 days, during which all patients will be washed out from their medications and put on venlafaxine. Limited dose of benzodiazepine (BDZ) will be allowed. The washout period may be shortened to allow a subject to enter the run-in phase as soon as the day after the start of the washout period.

During the 8-week run-in phase, eligible subjects will be treated with an open-label, flexible-dose of venlafaxine (a relatively new antidepressant with demonstrated efficacy, which is already in widespread clinical use [11-13]), administered once daily in a dose range of 75 to 225 mg to identify a dose that will achieve control of the subject’s acute symptoms. Only subjects capable of maintaining a stable dose regimen in the last week of this phase, and who also meet criteria of responder (a 50% reduction in the HAM-D_17_ score from baseline at two consecutive visits, and a score ≤12 at the last valid visit prior to the stabilization phase) will be eligible to enter the stabilization phase.

During the 6-week stabilization phase, subjects will continue to receive the fixed dose of venlafaxine established at the end of the run-in phase, and the dosage of venlafaxine cannot be adjusted. Evaluation visits will occur every 2 weeks. Subjects who maintain a stable dose regimen (dose unchanged) during this phase and meet criteria of remission (a HAM-D_17_ score ≤7) will be eligible to be randomly assigned to the single-blind phase of the study.

Subjects who complete the run-in and stabilization phases of the study and who meet prospectively defined criteria will be randomly assigned, according to permuted block design with a fixed block, in a 1:1:1 ratio, to one of three groups: group 1 on active rTMS and venlafaxine; group 2 on sham rTMS and venlafaxine; and group 3 on venlafaxine alone, where responders will be maintained on the same effective dose of venlafaxine for the entire duration of the randomized controlled trial (RCT), unless they relapse and will have to exit the protocol and enter a naturalistic follow-up. Subjects will be treated until they meet either prospectively defined criteria for relapse(HAM-D_17_>12, reduction in the HAM-D_17_ score ≤50% from baseline, and meeting DSM-IV criteria for a diagnosis of MDD) or predefined study conclusion criteria.

#### Interventions

For rTMS, a 100mm figure-of-eight-shaped coil and a MagPro Compact stimulator (Dantec Company, Copenhagen, Denmark) will be used. The resting motor threshold (RMT) will be recorded daily, and defined as the intensity required to elicit at least five motor evoked potentials (MEPs) of 50 μV peak-to-peak amplitude in ten consecutive stimulations, when the coil is placed over the left primary motor cortex (the site for stimulation will be 5 cm anterior to, and in the same parasagittal plane as, the site for maximal stimulation of the abductor pollicisbrevis). The parameters used for rTMS delivery will be: 90% of RMT; 10 Hz; 5 minutes; 6 seconds per train; 24 seconds inter-train interval; 1,200 pulses per day. In maintenance phase, each participant will receive two sessions per week for 4 weeks (1 month), followed by two sessions every 2 weeks for the next 4 weeks (1 month), and then two sessions per month for 10 months.

Sham stimulation will be given at the same site and frequency, using a Magstim sham-coil system (Whitland, UK). During rTMS, participants will be instructed to keep their eyes open and relax.

All subjects will be given general counseling by appropriately trained and experienced doctors once per month. A 'best guess’ questionnaire will be given to the patients at the end of the study, to assess whether they were blinded to the intervention applied.

#### Outcomes

The primary efficacy criterion will be the time to first relapse during the single-blind phase. Secondary efficacy criteria will include: a reduction in the HAM-D_17_ score; an improvement of global clinical status assessed by the Clinical Global Impression of Severity for Depression (CGI-S-DEP); and a change in subject functioning determined by the Personal and Social Performance (PSP) scale. Suicidality will be assessed by the Columbia Suicide Severity Rating Scale (C-SSRS). An independent evaluator, separated from the treatment team, will administer rating scales.

#### Statistical analysis

Relapse will be estimated by the Kaplan–Meier method. Time to relapse will be summarized (number of events, number of censored subjects, median, and 25th and 75th percentile of time to events) and treatment differences will be compared using a two-sided log-rank test. The estimate of the hazards ratio and its 95% confidence interval will be based on the Cox proportional hazards model with treatment as the only covariate. Secondary efficacy variables will include the HAM-D_17_, CGI-S-DEP, and PSP scores. The overall significance level across treatment groups for all secondary analyses will be 0.05 (two-sided) with no multiplicity adjustments.

## Discussion

MDD is a condition that is difficult-to-treat, and is predicted to rank second on the list of 15 major diseases in terms of burden in 2030 [14], largely due to its highly recurrent nature. The characteristics of the treatment process for depression include acute phase treatment, a consolidation treatment period, and maintenance therapy. Inadequate treatment will result in recurrence; therefore, clinicians must be prepared to manage relapse and recurrence aggressively throughout all phases of treatment. However, even if antidepressant medication therapy is strictly followed, many individuals with depression still suffer from recurrence.

rTMS has been approved as an effective add-on therapy for MDD [15,16], and is known as one of 'the four brain science technologies of the 21^st^ century’ along with functional magnetic resonance imaging (fMRI), positron emission tomography (PET), and magnetoencephalography (MEG). rTMS has a favorable tolerability profile, and was approved by the US Food and Drug Administration (FDA) in 2008, and by the European Union (EU) in 2012, for the treatment of patients with medication-refractory unipolar depression. At present, in ClinicalTrials.gov, there are 102 clinical studies worldwide assessing the use of rTMS in depression. However, most of these are focusing on acute phase and consolidation treatment, with only a small minority of studies investigating rTMS use for maintenance treatment and recurrence prevention in depression.

Although some research has indicated that rTMS may be effective in the prevention of MDD recurrence, these conclusions are preliminary, with most descriptions of the maintenance effects of rTMS being case reports that are limited by their short-term nature. For example, a case series suggested beneficial effects in 8 of 11 patients with refractory depression, who maintained responder status over a period of 3 months with rTMS therapy [9]. A recent study conducted by Janicack *et al*. examined the persistence of rTMS effects in a 24-week follow-up, and suggested that the therapeutic effects of TMS were durable, and that TMS may be successfully used as an intermittent rescue strategy to preclude impending relapse [17]. Their work describes the results of a clinically plausible, effective, and safe strategy for maintenance of acute benefit in clinical practice, and provides a framework for the design of future controlled trials assessing rTMS for maintenance or relapse prevention. However, rTMS was not applied in the maintenance phase of MDD in their study. Thus, we aim to use a RCT with a larger sample size to investigate if the rTMS treatment paradigm shows potential as an add-on treatment to antidepressant monotherapy for relapse prevention, and whether it might serve a useful future role as an option for maintenance treatment.

We expect to complete recruitment by December 2013, and anticipate that the results from this study will not only have implications for healthcare resourcing, but also facilitate improvements in the treatment of patients with depression. The results of this study will also allow us to determine if rTMS is an effective add-on intervention to antidepressants in the maintenance stage of MDD. These results may have important implications for the relief of the disease burden caused by MDD relapse.

Randomization and a single-blind design will be used in this trial to ensure adequate concealment. To date, this trial is one of the largest RCTs assessing the efficacy of rTMS for maintenance treatment of MDD. The trial is still in the recruitment phase.

## Trial status

Patient recruitment is still ongoing.

## Abbreviations

BDZ: Benzodiazepine; CGI-S-DEP: Clinical Global Impression of Severity for Depression; C-SSRS: Columbia Suicide Severity Rating Scale; DSM-IV: Diagnostic and Statistical Manual of Mental Disorders, fourth edition; ECG: Electrocardiography; ECT: Electroconvulsive therapy; EU: European Union; FDA: Food and Drug Administration; fMRI: Functional magnetic resonance imaging; HAM-D17: 17-item Hamilton Rating Scale for Depression; MDD: Major depressive disorder; MEG: Magnetoencephalography; MEP: Motor evoked potential; PET: Positron emission tomography; PSP: Personal and social performance; RCT: Randomized controlled trial; RMT: Resting motor threshold; rTMS: repetitive transcranial magnetic stimulation.

## Competing interests

The authors declare that they have no competing interests.

## Authors’ contributions

QT conceived the study. HW and YX designed the study protocol and wrote the manuscript. QT, LZ, and JG sought ethical approval. YZ, HW, RZ, and YC are responsible for the statistical analyses. The authors are the coordinators of the clinical centers that will enroll the trial participants. The corresponding author had final responsibility for the decision to submit for publication. All authors read and approved the final version of the manuscript.
